# Risk factors for breast cancer among women in Freetown, Sierra Leone, 2017: a case-control study

**DOI:** 10.11604/pamj.2024.47.184.34179

**Published:** 2024-04-15

**Authors:** Philip Pelema Gevao, Adel Hussein Elduma, Ernest Kenu

**Affiliations:** 1 College of Medicine and Allied Health Sciences, University of Sierra Leone, Freetown, Sierra Leone,; 2 Sierra Leone Field Epidemiology Training Program, Freetown, Sierra Leone; 3 University of Ghana, Accra, Ghana

**Keywords:** Breast cancer, socio-demographic risk factors, reproductive risk factors, behavioral risk factors

## Abstract

**Introduction:**

breast cancer is the most commonly diagnosed malignancy and an important cause of cancer death among females worldwide. The disease accounted for 25% (1.67 million) of new cancer cases and the fifth cause of cancer deaths. Incidence of all types of cancers is approximately 25% in Sierra Leone. However, there was no documented evidence on risk factors for breast cancer among women in the country. The main aim of this study was to assess risk factors associated with breast cancer among women screened for breast cancer in Freetown Sierra Leone.

**Methods:**

we conducted a case-control study on breast cancer involving 116 confirmed breast cancer cases and 116 controls. Questionnaire was designed to collect data on socio-demographic, reproductive and behavioral risk factors. Analysis was carried using logistic regression to assess the associations between breast cancer and the risk factors.

**Results:**

in the final multiple logistic regression, had formal educational level, (aOR 0.1, 0.03-0.26, p= 0.001) physical activity for more than 30 minutes per week (aOR 0.5 (0.9- 0.7, p=0.001). Cigarette smoking (aOR 4.8, 1.2-18.5, p=0.023) and family history of breast cancer (aOR 9.9 cigarette smoking (OR 4.4, 1.2-18.5, p=0.023) and family history of breast cancer (OR 9.9, 2.7-36.45, p=0.040) were identified as the main risk factors for breast cancer. This study did not find any statistically significant associations between reproductive risk factors and breast cancer.

**Conclusion:**

risk factors for breast cancer among women in Sierra Leone include educational level, physical activity, cigarette smoking and family history of breast cancer. We recommended screening program for women above 40 years and had history of breast cancer. Also, to establish breast cancer registry.

## Introduction

The breast serves as a very important function in a woman´s sexuality and also serves as a symbol of beauty. Therefore, when a woman observes changes in her breast, she becomes worried about the disfiguration and loss of sexual attractiveness, or even fear of death. Breast cancer is the most common malignancy and invasive cancer affecting 12% of women worldwide [1]. According to the World Health Organization, there were 2.3 million women diagnosed with breast cancer and 680000 deaths in the global. Breast cancer is the most prevalent cancer, where 7.8 million women were diagnosed with this cancer in the past 5 years by 2020 [2]. Even though the exact etiology of breast cancer is not fully known, however, a range of breast cancer risk factors have been recorded, along with; aging, oral contraceptive use, physical inactivity, too much consumption of alcohol, parity among others [3]. Breast cancer is a condition that is attributed to increasing age. It is known that for every ten years, the risk for breast cancer doubles until menopause when it declines, and age is considered as one of the leading causes of breast cancer [4]. Higher education level serves as a very important constituent, and an indirect reflection of socioeconomic status. Education tends to have an influence on breast cancer because women who are educated have fewer children, late age at child birth and utilize family planning methods [5]. Hereditary factors possibly will contribute in affecting some proportion of early onset breast cancer in different part around the globe; however, the role of hereditary factors cannot by itself be the sole reason for universal differences in risk. Studies done in Sweden, showed alterations in the BRCA genes were seen more among cases with a close relative with cancer of the breast, as opposed to those with no positive family history [6]. Age of menarche is considered as a risk factor for breast cancer. A case control study done in Morocco showed that females who start menstruation early had an increase in odds of developing breast cancer, as opposed to those who menstruate at a late age. This relationship was significant and observed among the age group 22 and 34 years [7]. The duration of breast feeding determines the risk of breast cancer. Study in Eastern India showed that women who had breastfed their children showed a low association with breast cancer compared with women who had not breastfed their children [3]. The risk of breast cancer is higher in women with no children or those having children very late in life. Studies done in Iran revealed that multiparous women had a very strong protective factor for breast cancer when compared with nulliparous women [8]. Other factors that might contributed in breast cancer are behavioral risk factors such as tobacco smoking, alcohol consumption, obesity, and physical activity [7,9,10]. According to the WHO records, Sierra Leone has an incidence 25.1% among the most common cancers in the country [11]. Risk factors like early menarche, late menopause, parity, breastfeeding, sedentary lifestyle, alcohol, etc., have been documented for breast cancer among women in other part of the world. However, in Sierra Leone, there is no documented evidence on risk factors for breast cancer among women. So, we conducted this study to assess the risk factors associated with breast cancer among women in Freetown, Sierra Leone.

## Methods

**Study design**: the research adopted an unmatched case-control study between January and July 2017.

**Study setting**: the study was conducted in Freetown, the capital city of Sierra Leone. The town comprises all the ethnic groups in Sierra Leone and the main ethnic groups are mainly the Mende, Temne, Limba, and Creole. Freetown is made up of Western area urban and Western area rural. The 2016 population and housing provisional census result estimate the population of Freetown to be 1,493,252 inhabitants, of which 1,050,301 inhabitants are in Western area urban and 442,951 in Western area rural (NS, 2016). The study was conducted in two breast cancer screening medical facilities, Well Woman Clinic located at number 9b Murray town road and Thinking Pink Clinic of number 11 Percival Street, Freetown, Sierra Leone. These facilities are the two largest medical facilities screening and managing cancer cases presently in Freetown.

**Study population**: the study population was all women between the ages of 20-70 years who were attending the Well Woman Clinic and Thinking Pink screening center in Freetown from January 2012

**Case definition**: case was defined as woman living in Freetown attending either Well Woman or Thinking Pink Clinic from January 2012 December 2016 between 20-70 years who had undergone screening for breast cancer with confirmed histopathology for breast cancer.

**Control definition**: control was defined as females between 20-70 years residing in Freetown attending Well Woman and Thinking Pink Clinics respectively for breast cancer screening and showed to be free from the disease after screening with the mammogram during the period January 2012 to December 2016.

**Exclusion criteria**: cases and controls, not residing in Freetown. Immobile, unconscious, and disoriented patients were excluded from control selection.

**Sample size determination**: to estimate the sample size for this study, stat calculator in Epi info version 7 was used. A sample size of (n) 116 breast cancer cases was calculated. It is assumed that 9% of the study population is exposed to tobacco smoking [12]. A detection of an odd ratio of 3 with a power of 80% is to be achieved with a 95% confidence level (5% significance). A ratio of 1: 1 cases and controls were factored in. Since the prevalence of a number of the known risk factors for breast cancer, such as age at menarche, were not available and prevalence of most risk factors for breast cancer has not been documented in Sierra Leone, the figure used as the least exposure prevalence was smoking, which was derived from the Sierra Leone Demographic Health Survey, 2013. For this study, a total of 116 cases and 116 controls were used.

**Sampling technique**: this study was carried out in two breast cancer screening centers in Freetown and patients with histopathologically breast cancer-positive cases were identified using five-year data records for breast cancer between the period from January 2012 to December 2016. A simple random sampling technique was used to identify cases for this study. All histopathologically confirmed breast cancer cases in Well Woman and Thinking Pink Clinics were included in the cases list. A total of 116 random numbers were generated using Stat Trek´s random number generator and 116 cases were selected from the list. Controls were selected among women attending the Well Woman and Thinking Pink Clinics in Freetown for breast cancer screening. Systematic random sampling method was used for control selection and 116 controls were selected.

**Data collection**: a questionnaire was structured based on the standardized questionnaire from the WHO Stepwise Approach to chronic disease risk factor surveillance (STEPS) and used for the data collection. The questionnaires were pretested at 34 Military hospitals in Freetown for reliability and efficiency before data collection was done. The questionnaire was administered through face-to-face interview and in their preferred language. Weight measurements were determined using a portable electronic (Secarobusta 813 extra-large digital reading) weighing scale calibrated to 0.1 kg and measures up to 150 kg. The height measurement procedure was carried out by asking the patient to stand against a wall and a point was marked at the top of the person´s head and measured using a tape measure in centimeters. Demographic variables were collected including age, sex, educational level, occupation, marital status and income information. Also, reproductive variables such as menarche, parity, menopause, contraceptive use, breastfeeding, family history of breast cancer were collected. Furthermore, behavioral variables including alcohol consumption, tobacco smoking, physical activity and obesity were collected.

**Data assurance quality**: proper quality assurance procedures and precautions were taken to ensure the reliability and validity of the data. The researcher selected two research assistants who had public health background and gave them adequate training. The principal investigator was part of the team during the interviews to ensure that relevant information in line with the objectives of the study was collected. The questionnaires were checked for mistakes and completeness before final entry into appropriate software for statistical analysis.

**Data analysis**: the raw data was entered into a Microsoft Excel spreadsheet for Windows 8. The data was cleaned, coded, and imported into STATA software version 14 for statistical analysis. Continuous variables were analyzed and presented as Mean ± standard deviation (SD). Categorical variables were presented as percentages. Bivariate analysis was conducted to determine the association between breast cancer (dependent) and the risk factors (independent variable). The odds ratio (OR) was computed and a statistical significance of 0.2 was as a cut-off point to include variables in the multivariate analysis. Variables that had a statistical significance in the bivariate analysis were entered into multiple logistic regression. Any variable in the multivariate analysis with a p-value less than 5 was considered a risk factor for breast cancer.

**Ethical clearances**: ethical clearance was obtained from the Government of Sierra Leone Ethics and Scientific Review Committee. Consent was sought from all the study participants and information provided for this study was handled with strict confidentiality.

## Results

**Demographic characteristics of respondents**: the study included a total population of 232 respondents comprising of 116 breast cancer cases and 116 controls. The majority of the controls were between the ages of 20-49 years whilst the cases were between 30-59 years. The mean age of cases and controls were 30.0±10.3 and 36.7±11.8 respectively. The ages of cases and controls are considered in regards to the level of education, 69.8: (81/116) cases and 95.7: (111/116) controls had some formal education whilst 30.2: (35/116) cases and 4.3: (5/116) controls had no formal education. 56.9: (66/116) cases and 58.6% (68/116) controls were married whilst 43.1: (50/116) cases and 41.4: (48/116) controls were single. Participants who were divorced or widowed at the time of the study were classified as single. Concerning the employment status of participants, 72.4% (84/116) cases and 83.6: (97/116) controls were employed and engaged in various activities whilst 27.6: (32/116) cases and 16.4: (19/116) controls were unemployed (Table 1).

**Table 1:** bivariate analysis of demographic, reproductive and behavioral factors of women breast cancer in Sierra Leone

Variables	Breast Cancer			
	**Cases**	**Controls**	cOR (95% CI)	P value
**Educational Level**				
Had formal education	81(69.8)	110(95.6)	0.1 (0.03-0.29)	<0.001
No formal Education	35(30.2)	5(4.4)	1.0	
**Marital Status**				
Married	66(56.9)	68(58.6)	0.9 (0.54-1.62)	0.791
Single	50(43.1)	48(41.4)	1.0	
**Employment status**				
Employed	84(72.4)	97(83.6)	0.5(0.26-1.02)	0.039
Unemployed	32(27.6)	19(16.4)	1.0	
**Age at menarche**				
> 13 years	50(43.5)	57(50.0)	0.8(0.44-1.34)	0.323
≤ 13 years	65(56.5)	57(50.0)	1.0	
**Age at menopause**				
> 50 years	14(28.0)	3(20.0)	1.6 (0.34-9.81)	0.536
≤ 50 years	36(72.0)	12(80.0)	1.0	
**Age at first birth**				
> 25 years	17(16.0)	18(22.5)	0.7(0.33-1.65)	0.426
15-25 years	89(84.0)	70(87.5)	1.0	
**Parity**				
Nulliparous	11(9.5)	27(23.3)	0.4(0.15-0.77)	0.005
Multiparous	105(90.5)	89(76.7)	1.0	
**Number of children**				
≥ 3	47(40.5)	53(45.7)	0.8(0.47-0.1.41)	0.43
< 3	69(59.5)	63(54.3)	1.0	
**History of breastfeeding**				
Never	8(7.0)	28(24.3)	0.2 (0.089-0.57)	<0.001
Eve	107(93.0)	88(75.7)	1.0	
**Oral Contraceptives**				
Ever	44(38.3)	47(40.5)	0.9 (0.52-1.60)	0.726
Never	71(61.7)	69(59.5)	1.0	
**Family history of breast cancer**				
Yes	22(19.0)	3(2.6)	8.8(2.5 1-47.04)	<0.001
No	94(81.0)	113(97.4)	1.0	
**Alcohol consumption**				
Ever	39(33.6)	32(21.6)	1.3 (0.73-2.42)	0.319
Never	77(66.4)	84(72.4)	1.0	
**Smoked cigarette**				
Ever	17(14.7)	3(2.6)	6.5(1.78-35.21)	0.001
Never	99(85.3)	113(97.4)	1.0	
**BMI (Kg/m^2^)**				
≥25	80(69.0)	82(70.6)	0.9(0.51-1.68)	0.775
<25	36(31.0)	34(29.4)	1.0	
**≥ 30mins physical activity/ week**				
Yes	66(56.9)	80(69.0)	0.6 (0.33-1.05)	0.05
No	50(43.1)	36(31.0)	1.0	

**Bivariate analysis**: participants with some levels of education were protected against the development of breast cancer and it was statistically significant at a 95% confidence level (cOR, 0.1; 95: CI: 0.03, 0.29). Women who were married had 7% smaller odds of developing breast cancer compared to single women. However, this result was not significant at a 95% confidence level (cOR, 0.9; 95: CI: 0.54, 1.62). Those women who were employed had 49% lower odds of developing breast cancer compared to those women who were unemployed and this result was not statistically significant (cOR= 0.5; 95% CI: 0.26, 1.02). The results showed that 43.5: (50/116) cases and 50.0: (57/116) controls had early menarche whilst 56.5: (65/116) cases and 50.0: (57/116) controls had late menarche. Women who had late menarche had 23% smaller odds of developing breast cancer compared to women who had early menarche. Women who had late menarche (> 13 years) were protected from the development of breast cancer in the bivariate logistic regression (cOR=0.77, 95% CI: 0.44, 1.34). Twenty-eight percent (14/116) of the cases and 20% (3/116) of the controls were late menopausal (>50yrs) while 72: (36/116) cases and 80: (12/116) controls were early menopausal (≤50yrs). These results indicated that participants with late menopausal status were about 1.6 times more likely to develop breast cancer compared to those with early menopausal status (cOR=1.6; 95% CI: 0.34, 9.8). Cases and controls representing 16.0: (17/116) and 22.5: (18/116) respectively had their first full-term pregnancy when they were 26 years or older whilst 84: (89/116) cases and 87.5: (70/116) controls had their full team pregnancy between age 15 to 25 years. Women who had their first full-term pregnancy when they were 26 years old had 26% smaller odds of breast cancer compared to women who had their full-term pregnancy between ages 15-25 years and this result was not statistically significant at 95% confidence level (cOR=0.7; 95% CI: 0.33, 1.65). The results indicated that 7: (8/116) cases and 24.3: (28/116) controls had never breastfed before whilst 93: (107/116) cases and 75.7: (88/116) controls had ever breastfed before and these groups were mostly the nulliparous women. The results showed that breastfeeding was a protective risk factor (cOR, 0.2; 95: CI: 0.08, 0.56). The results showed that 38.3: (44/110) cases and 40.5: (47/116) controls had ever used oral contraceptives while 61.7: (71/116) cases and 59.5: (69/116) controls had never used oral contraceptives. The use of oral contraceptives was not associated with the development of breast cancer (cOR=0.9; 95% CI: 0.52, 1.60). The results showed that 40.5: (47/116) cases and 45.7% (53/116) controls had less than 3 children whilst 59.5% (69/106) cases and 54.3% (63/89) controls had at least 3 children. It was observed that women who had at least 3 children had 19% lesser odds of breast cancer compared to those women with less than children (cOR, 0.8; 95: CI: 0.47, 1.41). The result shows that 19.0: (22/116) of cases and 2.6: (3/116) controls had a positive family history of breast cancer whilst 81.0: (94/116) of cases and 97.4: (113) of controls had no family history of breast cancer (cOR= 8.8; 95% CI: 2.51, 47.04).

In lifestyle risk factors results showed that out of 232 participants, 33.6: (39/116) cases and 21.6: (32/116) controls had ever consumed an alcoholic beverage before in their lifetime while 66.4: (77/116) cases 72.4: (84/116) controls had never consumed alcoholic beverage. The results showed that alcohol consumption was associated with the development of breast cancer. Women who consumed alcohol were 1.3 times more likely to have breast cancer comparing those who have ever consumed alcohol (cOR=1.3; 95% CI: 0.73, 2.42). Out of 232 participants, 14.7: (17/116) cases and 2.6: (3/116) controls smoked cigarettes while 85.3: (99/116) cases and 97.4: (113) controls had never smoked in their lifetime. Women who smoked had 6.5 times greater odds of developing breast cancer compared to those women who had never smoked (cOR= 6.5; 95% CI: 1.9, 35.2). Forty-three point one percent (43.1% (50/116)) cases and 31.0% (36/116) controls had not been actively engaged in various kinds of physical activities continuously at least twice a week in the last 6 months while 56.9: (66/116) cases and 69.0: (80/116) controls had engaged in some form of physical activity at least twice a week in the last 6 months. Physical activity was found to be protected against breast cancer in the bivariate analysis (cOR=0.6; 95% CI: 0.33, 1.05). The mean BMI of cases was 28.4±6.1 Kg/m^2^ and that of controls was 27.7±5.9 Kg/m^2^. BMI was found not to be associated with the development of breast cancer in the bivariate logistic regression at a 95% confidence level (cOR= 0.9; 95: CI: 0.501;1.68) (Table 1).

**Multivariate analysis**: factors had statistically associated in multivariate analysis included, having formal education (aOR= 0.1; 95% CI: 0.03, 0.3). The odds of breast cancer among those who practice 30 minutes of physical activities per week was 0.5 times compared to those who did not practice physical exercise (aOR 0.5 (0.9- 0.7, p=0.001). The odds of breast cancer among those who smoke cigarettes was 4.8 times as compared to non-smokers (aOR= 4.8; 95% CI: 1.2, 18.5). The odds of breast cancer among those with a breast cancer family history was 9.9 times as compared to those without a breast cancer family history (aOR= 9.9; 95% CI: 2.7, 36.5) (Table 2).

**Table 2:** multivariate analysis for the main risk factors of women breast cancer in Sierra Leone

	Breast Cancer		Unadjusted Odd ratio		Adjusted Odd ratio	
Variable	Cases	Controls	(95% CI)	P value|	(95%CI)	P value
**Educational Level**						
Had formal education	81(69.8)	110(95.6)	0.11 (0.03-0.3)	<0.001	0.09 (0.03-0.3)	0.001
No formal Education	35(30.2)	5(4.4)	1.0		1.0	
**Employment status**						
Employed	84(72.4)	97(83.6)	0.51 (0.3-1.02)	0.039	0.76 (0.4-1.61)	0.142
Unemployed	32(27.6)	19(16.4)	1.0		1.0	
**Family History of Breast cancer**						
Yes	22(19.0)	3(2.6)	8.82(2.5-47.04)	<0.001	9.9 (2.7-36.5)	0.001
No	94(81.0)	113(97.4)	1.0		1.0	
**Smoked Cigarette**						
Ever	17(14.7)	3(2.6)	6.46 (1.8-35.2)	0.001	4.8 (1.2-18.5)	0.023
Never	99(85.3)	113(97.4)	1.0		1.0	
**≥ 30mins Physical Activity/Week**						
Yes	39(33.6)	10(8.6)	0.59 (2.4-12.7)	0.05	0.52 (1.9-16.7)	0.04
No	77(66.4)	106(91.4)	1.0		1.0	

## Discussion

This present case-control study was aimed at evaluating the role of socio-demographic, reproductive and behavioral risk factors of breast cancer among women in Sierra Leone. The result of this study showed that there was statistically significant association with the development of breast cancer between educational level, physical activity, cigarette smoking, education may possibly affect breast cancer because women who are educated have fewer children, late age at child birth and utilize family planning methods and have higher risk of breast cancer [13]. In contrast to this however, the findings of this study showed that participants with some levels of education were protected against the development of breast cancer and was statistically significant in both bivariate and multiple logistic regression analysis. These findings may be due to the fact that educated women have increased awareness on breast cancer risk factors and they also seek early detection. Secondly, educated women are better informed about the benefits of regular physical exercise and the negative effect of cigarette smoking and alcohol intake. Positive family history was strongly associated with a nine-fold greater odds of developing breast cancer compared to those with no family history of breast cancer. This finding agrees with a population-based study carried out among women 40-74 years in the showed that women with first and second-degree relative with family history of breast cancer had a higher proportion of breast cancer [14]. The possible reason for this could be due to the fact that the study participants are genetically predisposed or maybe because of shared cultural beliefs. This study however, disagrees with a case control study on risk factors associated with family history of breast conducted in Pakistan, in which there was no association between risk of breast cancer and family history of breast cancer [15].

The current result showed that women who had ever smoked cigarette had higher odds of developing breast cancer compared to women who had never smoked. This result agrees with prospective cohort study conducted in USA found out that smoking status was absolutely linked with risk for breast cancer [16]. Another large prospective cohort study reported that beginning smoking before menopause and mainly before first full-term pregnancy was most strongly associated with increased possibility of breast cancer [17,18]. Physical activity was found to be protected against breast cancer. This study shows that women who engaged in physical activity for more than 30 minutes per week had 41% lesser odds of developing breast cancer compared to women who did not engage in physical activity. This finding in consistent with systematic review which reported strong relationship between breast cancer risk and reduction in the rate of physical activity [19]. in Nigeria found out that majority of women with breast cancer had little or no exercise before their diagnosis. They therefore concluded that physical exercise has no effect on the risk of breast cancer [20].

Considering all the reproductive risk factors for breast cancer in the final multiple logistic regression analysis, none was a significant predictor of breast cancer. The reason for this failure of the reproductive variables that were significant in the bivariate analysis especially breastfeeding and parity to arrive at statistical significance in the multiple logistic regression may be as a result of collinearity. different studies found some significant associations between breast cancer and reproductive risk factors, and a high degree of collinearity as was expected between these variables. The synergistic role of breastfeeding and pregnancy and breastfeeding and number of children on risk factors for breast cancer shows that the combine effect of breast feeding and pregnancy were stronger than the effect of pregnancy alone. There was also a strong univariable association with number of children attenuated in the multivariate logistics analysis once breastfeeding was controlled for, and much change was not seen with breastfeeding. They came to the conclusion that these observations cannot be attributed to collinearity alone, but number of children was probably confounded by the effect of breastfeeding. The findings of this study also agree with similar studies conducted in Nigeria where apart from age at first full-term pregnancy, all the other reproductive variables that were considered failed to show any statistical significance in the multiple conditional logistic regression model and this failure was due to collinearity between age at first full-term pregnancy and the other reproductive variables [21]. With respect to the use of oral contraceptive pills, this current study showed that the use of oral contraceptive pill was not associated with the development of breast cancer among women and this association was also not statistically significant. In contrast to these findings, case-control study conducted in the Morocco suggested that oral contractive use above 6 months is linked with an increased risk of breast cancer [22].

## Conclusion

Risk factors associated with women breast cancer in Freetown were individuals with no formal education, family history of breast cancer, smoking cigarette and individuals with no physical activity more than 30 minutes per week. We recommended policy makers to be alerted in order to control programs to minimize the incidence of breast cancer in Sierra Leone. Screening programs to be developed by the Ministry of Health and Sanitation, and all women above 40 years especially those with a positive family of breast cancer to be encouraged to undergo screening for breast cancer. Also, to set up a breast cancer registry, and develop preventive, diagnostic, treatment guidelines and palliative care.

### What is known about this topic

- *Breast cancer is a major health problem for women globally;*
- *Risk factors such as early menarche, late menopause, parity, breast-feeding, sedentary lifestyle, alcohol have been proved to have association with breast cancer*.

### What this study adds

- *This is the first study to address the risk factors of breast cancer in Sierra Leone;*
- *We identified individuals with no formal education, family history of breast cancer, smoking cigarette and individuals with no physical activity more than 30 minutes per week as main predictors for women's breast cancer*.
